# The role of vocal individuality in conservation

**DOI:** 10.1186/1742-9994-2-10

**Published:** 2005-06-16

**Authors:** Andrew MR Terry, Tom M Peake, Peter K McGregor

**Affiliations:** 1 IUCN – The World Conservation Union, Regional Office for Europe, Boulevard Louis Schmidt 64, 1040 Brussels, Belgium; 2 127 Kennington Avenue, Bishopston, Bristol BS7 9EX, UK; 3 Duchy College, Stoke Climsland, Callington PL17 8PB, UK

## Abstract

Identifying the individuals within a population can generate information on life history parameters, generate input data for conservation models, and highlight behavioural traits that may affect management decisions and error or bias within census methods. Individual animals can be discriminated by features of their vocalisations. This vocal individuality can be utilised as an alternative marking technique in situations where the marks are difficult to detect or animals are sensitive to disturbance. Vocal individuality can also be used in cases were the capture and handling of an animal is either logistically or ethically problematic. Many studies have suggested that vocal individuality can be used to count and monitor populations over time; however, few have explicitly tested the method in this role. In this review we discuss methods for extracting individuality information from vocalisations and techniques for using this to count and monitor populations over time. We present case studies in birds where vocal individuality has been applied to conservation and we discuss its role in mammals.

## Review

### Introduction

Signals can contain information useful to conservation [1,2]. Until recently, communication behaviour had a limited role in conservation, being restricted to enhancing captive breeding programs [3] or use in species counts [4]. However, knowledge of how individuals within a population communicate and what they are communicating can generate information ranging from measures of habitat use to genetic fitness [2,5] that can be applied to conservation and that may neither be possible nor desirable to extract using other methods. In this review we shall concentrate on a subsection of communication behaviour that underlies most attempts to gain useful information from signalling, namely individuality. We discuss the different applications and types of information that can be extracted using vocal individuality. Further, we consider the different methods currently applied and the results gained with different taxonomic groups. Finally, we discuss some of the limitations and future directions that this technique can take. As a consequence of our area of research we have concentrated our discussion on the role of acoustic vocal individuality in conservation, although the principles involved equally apply to other signalling modalities, with the possible exception of chemical signals where the techniques may currently be lacking.

#### Vocal individuality

A pre-requisite for discrimination and individual recognition is that, in the signals being used, there should be low within-individual variation and high between-individual variation [6,7]. Many studies have shown the presence of individually distinctive vocal features in a wide range of animal species and it seems that vocal individuality is most likely a feature of all vocally active species and is caused by a series of genetic, developmental and environmental factors [8,9]. The level of individuality and the difficultly in extracting and using it will differ between species and we discuss these issues later in the review (also see [10-12]).

When discussing the uses of vocal individuality we distinguish between two commonly confused terms; discrimination and identification. Discrimination requires that individuals differ enough at one point in time to be separated. Identification requires that an individual's vocal features remain constant enough to be associated with that individual for periods of time. Identification is harder to demonstrate and has more useful conservation applications than discrimination [2,13]. Discrimination is limited to census tasks, but identification allows individuals to be monitored over time, which can yield useful life-history information [2,13]. It is possible to have discrimination without identification but not *vice versa *and the extent to which individuals can be identified should be carefully considered in any cost/benefit analysis of using vocal individuality as a monitoring tool.

#### Vocal individuality and conservation

Although several studies have suggested that vocal individuality could have a role as a useful conservation tool [5,14,15], it has been rarely used or tested in census or monitoring roles (some examples of use and testing are given below). In the following sections we discuss some of the different aspects of conservation biology where the identification of individuals can either yield useful data or be used as a non-invasive marking technique that may offer an alternative to invasive approaches such as mark-recapture or tagging.

##### Conservation models and identifiable individuals

Several studies by Sutherland, Goss-Custard and colleagues [16-20] have shown the importance of individual differences in predictive models. Assumptions of individual equality have largely come from difficulties associated with collecting and then modelling the baseline demographic data [21]. In most cases individual identification is required to extract this demographic data.

Population viability analysis (PVA) is the most widely used form of conservation model. These models attempt to predict the potential fate of a population given a set of demographic and environmental parameters. PVA models include demographic stochasticity, that is, fluctuations in population size due to random individual differences [22], as a form of 'sampling error' [23]. Mean expected survival and fecundity are estimated from a limited data set and made equal for all individuals; stochasticity is included as the level of variance around this mean when survival is calculated for the model population [23,24]. Increasing the variance increases the risk of extinction faced by the population. However, these assumptions are biologically untenable. The inclusion of non-random individual variation acts to reduce the effects of demographic stochasticity, leading to the result that the predicted extinction risk faced by populations may be overly pessimistic [23,24]. By identifying individuals, it is possible to generate more accurate predictions concerning the nature of individual variation in parameters such as fecundity and survival, which will increase the accuracy of predictions of PVA models [17,23].

##### Behavioural traits

As well as affecting large-scale population models, identifying individuals can highlight behavioural traits that may affect the conservation value (in terms of effort and resources) of different subsections of a population [5]. In many species there are large asymmetries in lifetime reproductive success, with only a few individuals contributing to the next generation. For example, 17% of a population of common buzzards (*Buteo buteo*), accounted for 50% of the following year's fledglings [25]. Other bird species have yielded similar values [26]. Kelly [27] showed that a few (8%) cheetah (*Acynonyx jubatus*) matrilineages produced over 50% of the population over a 20-year period, and studies in other mammals have yielded equally high differences in reproductive success [28,29].

Individuals within populations also differ in whether they are residents or floaters. Floating individuals often occupy lower quality habitat, possibly representing 'sink' populations, and generally do not breed or breed with lower success [30-32]. This difference in breeding status has obvious implications for management strategies. Monitoring fluctuations in the proportion of floating individuals may also be a more revealing indicator of habitat quality [5]. Also, if translocation is considered as a management option, floating individuals may be suitable for translocation [33], as they may show reproductive success equal to other residents when moved to suitable habitat [30].

##### Sampling bias

In most cases it is not possible or desirable to count an entire population; usually, census techniques take samples from a population and use them to estimate the true population size [34,35]. All sampling techniques make assumptions about the populations they are counting and violation of the assumptions leads to biases in the estimate. Unless accounted for, biases can be pervasive and generate misleading census estimates. Accounting and testing for bias within a census can be difficult without either a complete knowledge of the population or a subset of identified individuals [34]. For example, the use of playback to elicit responses from individuals in acoustic surveys can cause a bias by only attracting certain individuals [36,37]. Using radio-telemetry, Conway et al [36] found both a seasonal and density-dependent bias in response to playback by Yuma clapper rails (*Rallus longirostris yumanensisi*). Playback elicited responses from 10–40% of males present. This also caused biases in the identification of preferred habitat for the rails [36]. A similar study with black rails (*Laterallus jamaicensis*) showed differential responses to playback due to factors such as breeding status, with non-nesting males being the most likely to respond [37]. Ogutu & Dublin [38] found playback to be an affective census tool for lion (*Panthera leo*) and spotted hyena (*Crocuta crocuta*) populations; however, placement of playback speakers near range borders instigated territorial disputes and prey abundance was also a significant source of bias.

Floating individuals can bias a census as they are usually more mobile than resident individuals. For example, floating male bitterns (*Botaurus stellaris*) were repeatedly counted in acoustic surveys causing a population of 15 individuals (nine residents and six floaters) to be counted as 25 [39]. This bias was detected by applying vocal individuality information to the population (further details below).

Sampling techniques that catch and mark individuals within a population are likely to also suffer from biases [5]. For example, the key assumptions of most mark-recapture approaches are that individuals are equally likely to be caught and that marks do not affect their subsequent behaviour and survival [34]. However, several studies have shown that capture techniques often only mark a subsection of the population (e.g. [40]). For example, radio-tracked corncrakes (*Crex crex*) called on 75–80% of nights during one breeding season and this was used to generate census guidelines for that species [41]. However, a subsequent study found a far lower calling rate (~ 40%) in a different population, which led to under-estimates of the population size [40].

##### Welfare considerations

Although marking techniques can allow the unequivocal identification of individuals, invasive marking of individuals can have both short-term and long-term effects on those individuals. Short-term effects include direct costs of the capture and handling process itself [5]. Longer-term costs include avoidance of the capture area, stress-related susceptibility to disease, increased susceptibility to predation and loss of subsequent reproductive success (e.g. [42]). Both short-and long-term effects of marking are contentious issues, with often equal numbers of articles showing the presence or absence of an effect. An example of this is the debate surrounding the causes of extinction of the Serengeti population of wild dogs (*Lycaon pictus*). The rapid spread of disease through the population was correlated with capture and handling [43]. However, recent analysis has shown that this relationship was likely to be non-causal and that the administered vaccines failed to take effect during an outbreak of the disease [44]. Another example comes from studies attempting to show effects of neckbands on survival in goose species [45-48].

It can be difficult to study the effects of capture and handling and the subsequent effects of marks on individuals unless there is an individually identifiable (or non-invasively marked) control group for comparison. For example, Nimon et al [49] used artificial eggs to non-invasively measure heart rates in Adélie penguins (*Pygoscelis adeliae*) and showed that handling individuals pre-disposed them to extreme reactions to human-induced stress [49,50]. However, it is safe to assume that the capture and handling of animals can only have deleterious effects, even if they may not be immediately obvious. Careful consideration has to be given to balancing benefits gained by invasive marking against potential adverse effects on the individuals or population. In our view, the default should be non-invasive marking techniques, such as vocal individuality – a view that has increasingly been adopted by journals in their recommendations to authors (e.g. [51]).

##### Life-history parameters

Techniques that identify individuals are used to generate life-history data, such as habitat use, survival, recruitment, immigration and emigration. This information can then be used to test hypotheses concerning the agents of decline facing a population or test the effects of management strategies [35,52]. Radio-telemetry is often considered the most productive monitoring technique. However, aside from concerns inherent in catching individuals and biases in data generated, radio-tags have a limited operational life, often of one year or less [53]. If vocalizations remain constant over time, they can provide a long-term monitoring option [53,54].

##### Summary

We have reasoned that vocal individuality should be widespread (if not ubiquitous) and can generate useful conservation information. Also, as a non-invasive marking method it may provide less biased data than other marking techniques and have fewer adverse welfare implications. We now discuss methods for extracting vocal individuality and some instances in which it has been applied to conservation questions in birds.

### Methods used in vocal individuality

The methods used to collect and analyse vocalizations will, in many cases, determine whether vocal individuality is used as monitoring tool. As the amount of equipment, analysis time and specialist knowledge required to discriminate between individuals increases, the likelihood of the technique being used decreases. Thus, in this section we discuss some general points concerning the collecting, processing and analysis of vocalizations for individuality.

#### Equipment and analysis

Before any statistical analysis takes place, there are a number of practical considerations concerning the process of converting an animal's vocalization into data points. Although some of these considerations may seem trivial, ignoring them can render the approach useless before it has started. This section highlights some of the points that we have encountered in our experience with sound recording and analysis; many texts (e.g. [56]) provide in-depth discussion of these topics.

The best type of microphone to use is determined by features of the vocal signal, predominantly its frequency range [57,58]. Shotgun and parabolic microphones are most commonly used for individuality studies and have different characteristics [57]. Parabolic reflectors amplify the signal entering the microphone and are more directional than shotgun microphones, reducing the reverberations in recordings [57]. However, parabolas cannot have a flat frequency response because the parabola diameter determines the extent to which each frequency is amplified and also determines the lowest frequency that can be recorded (e.g. [57,58]). Species with low frequency vocalizations, e.g. European bitterns (frequency range 100–200 Hz) should be recorded with a shotgun microphone with a flat response in this frequency range.

Similarly, the best type of recorder to use is determined by its ability to capture the sounds of interest with the necessary temporal and frequency resolution. Manufacturers' performance specifications are a starting point, although these may often be optimistic, subject to change with age of the machinery and may differ between individual recorders of the same model (particularly analogue tape recorders). It is therefore advisable to include a calibration sound of known frequency and duration at the beginning of a recording session. Digital recorders (solid-state or DAT) are limited by the sampling rate of their analogue to digital converters, so special care is necessary when recording high frequency sounds. Increasingly, field recordings are being made with devices using data compression to increase storage capacity (e.g. MiniDisc). The data compression algorithms remove parts of the signal that are not perceptible to humans, but they can also remove or distort important biological signal components [57].

When recordings are being made, the record level must be adjusted to prevent the signal becoming overloaded and the consequent introduction of distortion. We recommend that the recordist adjusts levels during recording as automatic gain controls rarely adequately control record levels of animal signals. In many cases it will be advisable to include a high-pass filter between the microphone and the recorder as this has the effect of removing low frequency background noise and signal strength is better represented by the recorder's level meters.

When analogue signals are digitised (either during recording or analysis) the highest frequency of interest must be less than half the sampling frequency (the Nyquist frequency), otherwise the higher frequencies will get mirrored in the new digitised signal [59-61]. Low-pass filtering should be used to remove sound from above the Nyquist frequency and filtering to remove unimportant parts of a recording is a method of minimising the amount of signal processing required [61].

**Figure 1:**
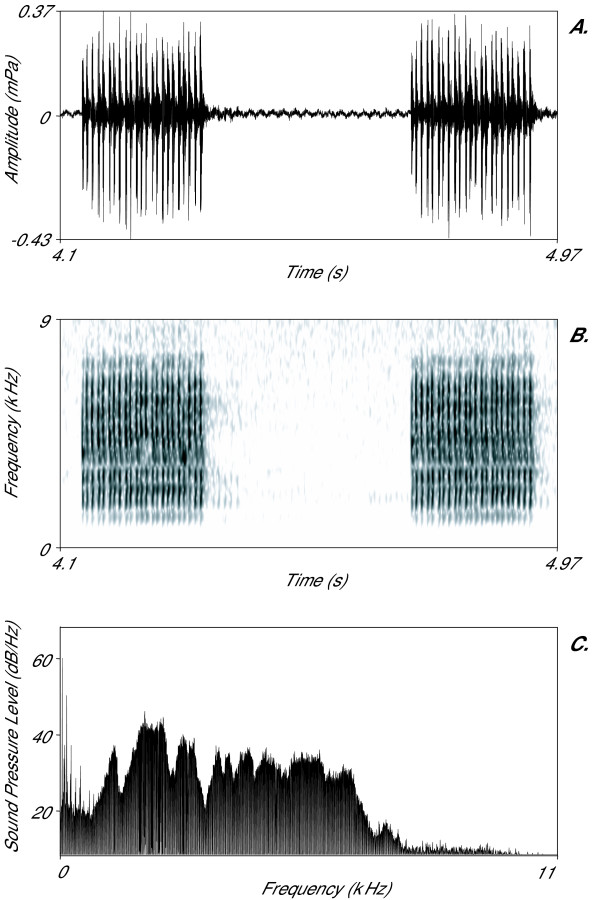
Three common forms of signal representation. An example of a corncrake call displayed as: (A) a waveform which plots temporal information on the *x *axis and amplitude on the *y *axis; (B) a spectrogram which plots temporal information on the *x *axis, frequency on the *y *axis and amplitude in the image greyscale; (C) a power spectrum, which plots frequency information on the *x *axis and sound pressure level on the *y *axis. The spectrogram was made with a 2 msec time step and a 20 Hz frequency step (Hamming window).

Once a signal has been digitised and filtered, it can be measured. Although complex signal processing, as well as automated measuring algorithms, are becoming increasingly common, most measures are taken from three forms of signal representation; waveform (Fig. 1A), power spectrum (Fig. 1B) and spectrogram (or sonagram, Fig. 1C). The waveform shows changes in amplitude (acoustic pressure) with time and is suitable for taking temporal measures. The power spectrum is generated from the waveform and shows changes in amplitude at different frequencies; this is suitable for measurements of features such as dominant frequency. A spectrogram is a three-dimensional display plotting frequency against time, with amplitude shown as intensity of grey scale or in colour. Spectrograms are very useful for visualising sounds and can be used in qualitative comparisons (e.g. [11,53]), but should not be used for direct measurements. This is because there is a fundamental trade-off when producing spectrograms between the resolution of time and frequency information; high resolution cannot be achieved in both features [62]. We suggest that waveforms should be used for temporal measures and power spectrum for frequency measures. Reported measurements should also always be accompanied by the minimum difference (the cursor increment) that could be measured in addition to resolution limitations imposed by the sampling frequency of A/D conversion.

#### Qualitative assessment of vocal individuality

Qualitative comparisons of sounds can be either visual, through graphic representations such as spectrograms, or aural through recordings or listening in the field. Aural identification is possible and field researchers with extensive experience of their study species can often identify individuals by ear [11]. However, there are few studies that address the use of aural comparisons as a census technique. Gilbert [63] showed that discriminating and identifying individual European bitterns by ear was affected by experience and that only small sample sizes could be discriminated.

More common is the use of visual representations of sounds such as spectrograms to discriminate between and to identify individuals [11,12,14,15,64-67]. In most cases, qualitative comparison is used as the first level of analysis, for example, in selecting sounds to be used in further quantitative tests [67]. In some cases qualitative comparisons have proved more effective than quantitative approaches [68]. Janik [66] compared several techniques used to discriminate between bottlenose dolphin (*Tursiops truncates*) signature whistles and found that visual comparison was the most effective at separating individuals.

The main advantage of qualitative assessment is that observers are capable of complex pattern recognition, perhaps using the overall 'gestalt' of an image in a comparison [2,65]. Features that may not be adequately represented in quantitative tests are considered in qualitative comparisons. Similarly, changes in small-scale measures can be weighted equally with large-scale features. The use of qualitative assessment contains several potential disadvantages associated with the subjectivity of human decision-making, which can lead to biases or inaccuracies [65,67,69-71]. It is possible to address these issues by measuring the repeatability of an observer's classifications; however, an observer with high repeatability may not make reproducible classifications [71]. It is possible to measure the degree of inter-observer reliability and use this to determine whether different observers can be used to classify the sounds (see [67] for discussion of inter-observer reliability scores).

#### Quantitative assessment of vocal individuality

Multivariate approaches are commonly applied to vocal individuality studies and are particularly suited to classification tasks [72]. They can be broadly divided into those that discriminate between individuals by finding differences between them and those that find similarities between them. We shall discuss each in more detail.

Discriminant function analysis (DFA) is a multivariate difference statistic commonly used to show which variables best discriminate between two or more groups. It does this by combining the variables with weighting coefficients to create a set of functions that can discriminate the groups. Once these functions are established, they can be used to classify new data into one of the pre-existing groups. This corresponds to the three main uses of DFA in vocal individuality; establishing variation, data reduction and classification. Establishing variation and paring down the parameters used are the first steps in any investigation into vocal individuality. Invariably many measures are taken from the vocalizations (often >20) and not all of them will be effective in discriminating individuals. For example, Gilbert et al [53] measured 23 features of bittern boom trains and used DFA to reduce these down to 7 features which allowed effective discrimination. A specialised form of this analysis is called stepwise discriminant analysis (SDFA) and it enters variables, one by one, into the analysis until there is no further increase in discrimination accuracy [73]. This can be a powerful way of extracting an 'optimal' subset of features and reducing further analysis time [72,73]. Once the discriminant functions have been established, they can be used to classify cases to the pre-existing groups. Measures of classification success (percent cases correctly classified) are often cited and are most often high (>80%). However, this measure does not test the functions' generality [73]. The discriminant functions must be validated with data not used in their creation. Most common statistics packages allow validation by not using some of the cases to produce the discriminant functions, and then classifying these unused data. This validation can either be a jackknife, where half the cases are left ungrouped and then classified, or a leave-one-out test where one case at a time is ungrouped and classified. Classification scores without any validation are almost meaningless in the context of their applicability to vocal individuality. Many studies have used DFA to show vocal individuality in avian [10-13,74,7], canid [78,79] and primate [80] species. Implicit in many of these studies is a potential use of DFA to generate conservation data. However, the question remains whether DFA on its own can generate useful conservation information. In reality the role of DFA classification in conservation is limited [2,81]. This is because in all published examples of the use of DFA for vocal individuality the type of DFA used can only classify vocalizations to particular individuals if all individuals are known; it cannot accommodate vocalizations from new individuals. This limitation is overcome by a non-parametric form of DFA known as adaptive kernel-based DFA, in which the range of values for inclusion into the kernel of existing groups is defined.

**Figure 2:**
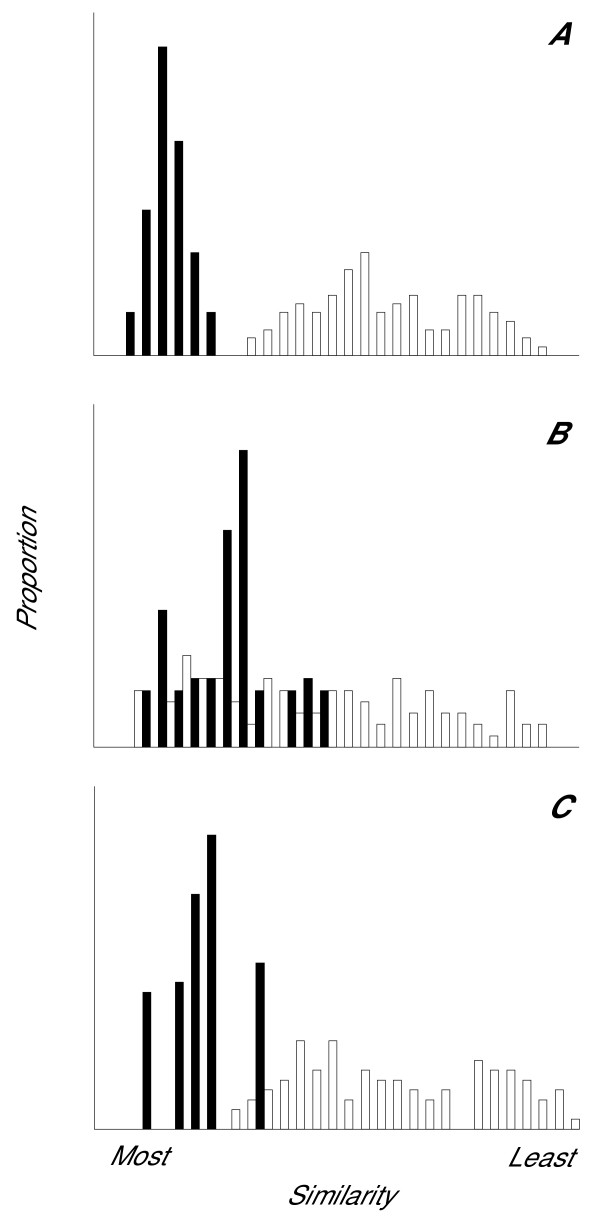
Examples of distributions of within-and between-individual similarity values. Distributions of within-individual (black bars) and between-individual (white bars) pair-wise comparisons in a similarity analysis. The ideal case (A) has no overlap between the two distributions; however, in cases of complete overlap (B) the technique becomes useless. The most common situation is one of partial overlap (C). The extent of this overlap can be used as a measure of confidence in the technique.

Similarity techniques offer a different approach to discrimination that avoids most of problems experienced when using DFA. They do not require complete knowledge of the population being monitored [2,81]. When using similarity techniques two cases are compared and if they are within a pre-defined threshold, they are classified as coming from the same individual. If a new individual joins the population, it should be outside the threshold for all known individuals. The two most commonly used approaches are acoustic space, which compares measures taken from vocalizations and cross-correlation which is used to compare sounds directly [2]. When a series of measurements are taken from a sound they can be used as coordinates that define the location of that vocalization in an acoustic space whose dimensions are determined by the number of variables [13,82]. The Euclidean distance between locations is used as the measure of similarity. This approach has been successfully used to identify individuals within and between seasons in several non-oscine bird species [13,53,83]. Cross-correlation is used to compare representations of whole sounds, most often these are spectrograms [59,84]. Two spectrograms are incrementally passed over each other and at each stage a Pearson correlation coefficient is calculated. The maximum correlation value is used as the similarity measure [84]. Many cross correlation routines move spectrograms only along the time axis and thus two sounds with identical frequency contours but centred on different frequencies would give a low similarity value. This is avoided by routines that move along time and frequency axes. The only conservation application of cross-correlation that we are aware of was to monitor wild turkey (*Meleagris gallopavo mexicana*) populations [85]. However, it has also been used show individuality [12,86-88], dialect differences [89], vocal learning and development [69,90] in several species of songbird. The advantage of cross-correlation is that it considers the entire sound objectively. However, care has to be taken with the particular sound types being compared, the amount of noise included in the signal and with the settings used to create the representations, as these factors will adversely affect the similarity value generated (for further discussion see [81,84,91]). Once a similarity measure has been used, the distributions of within-individual and between-individual similarity values can be compared (see Fig. 2). With an ideal similarity measure, the two distributions would not overlap, i.e. there would be no chance of making a false identification or discrimination [13]. However, in reality a certain amount of overlap will always occur and the extent of this overlap can be used to show how effective the technique will be (see Fig. 2 and [2,81,92]). Note that it will be impossible to maximise correct identifications and simultaneously minimise false identifications. However, studying the area of overlap can aid the setting of a threshold.

A number of complex non-linear models used for speech recognition have also been applied to bioacoustic signal analysis, and may represent the future direction for analysis tools as researchers try to compare increasingly complex signals. Models that have been used in bioacoustic studies include dynamic time warping [93-95], artificial neural networks [96-98], hidden Markov models [95] and linear predictive coding. These models can be divided into those that function by modelling sound production (linear predictive coding and hidden Markov models) or sound perception (neural networks). Currently, the most commonly used of these models are artificial neural networks. Originally designed as models of biological neural networks, they contain a network of inter-connected simple processing units that work in parallel to solve complex classification tasks [99,100]. The connections between the units are weighting coefficients. Neural networks solve classification problems by iteratively adjusting the weighting coefficients and combining them with the parameters until some pre-determined classification error value is achieved. The combination of simple algorithms used to classify highly optimised parameters makes them powerful and versatile tools. Neural networks have several advantages over other techniques. First, they are non-linear and can work with data that cannot be separated with linear classification tools [100,101]. Second, there is a huge range of different neural network types, and they have been applied to many classification and regression tasks. This provides a large source of reference material to draw on [98]. Third, they can be used to find clusters of similar vocalizations, set up groups based on those clusters and then classify new data to one of these groups or create a new group (e.g. Kohonen networks [102]). Neural networks have successfully identified individuals from their vocalizations in tungara frogs (*Physalaemus pustulosus*) [103,104], fallow deer (*Dama dama*) [96,105], Gunnison's prairie dog (*Cynomys gunnisoni*) [106], Stellar sea lions (*Eumetopias jubatus*) [97] and killer whales (*Orcinus orca*) [107]. Elsewhere we have shown that different neural network models were able to accurately count and identify individual corncrakes in a series of simulated census and monitoring tasks [98]. These types of model make it possible to create automatic analysis and identification systems [95], which will reduce analysis time, increase the amount of data that can be analysed and make complex classification techniques more available. All of these factors will be important in any conservation application. Note that, as with any statistical tool, they cannot be treated as complete black boxes, and some care has to be taken to adhere to the assumptions or limitations of the models (e.g. neural networks: [101], hidden Markov models: [95]).

### Three case studies

Here we detail three cases where vocal individuality has been used to obtain conservation information on bird species or where the potential for generating such information has been investigated. Vocal individuality has been used to correct for census error and bias in bitterns and corncrakes, to follow habitat use in corncrakes and owls, to monitor annual turnover in bitterns and owls and to produce data on which to run survival models in bitterns.

#### European bittern

The bittern is IUCN red-listed and is strictly protected under the EU Birds and Habitats Directives due to large population declines (>50%) in the last 25 years [108]. Bitterns are found in dense reed-bed habitat and are secretive, making monitoring difficult [11]. The use of leg rings can be a useful method to identify individuals; however, bitterns are rarely seen or caught and the only practical census and monitoring method involves counting calling males [109]. Male bitterns have an individually distinctive long-range vocalization [11,65] – the boom – and booms are used to census and monitor individuals. The bittern is currently the only species we are aware of that is routinely counted and monitored using vocal individuality. Bitterns have been monitored in the United Kingdom in this way for over 10 years, allowing long-term population data to be extracted [53]. Gilbert et al [53] identified males between years by their booms and used these data as the basis for a survival analysis and to monitor movements between populations for the UK populations (although see [12], for possible differences with Italian populations of bitterns).

Terry [110] developed a semi-automatic method that discriminated between relatively large numbers (>40) of bitterns and could also extract the discriminating measures from poor quality recordings. The main discriminating feature was the dominant frequency of the boom and this was found to vary little over very short periods of time (days). However, recordings made between years of four radio-tagged individuals [53] showed within-individual changes over time in the dominant frequency that sometimes exceeded between-individual differences. Bitterns could be identified from year to year by taking several measures of the boom manually [53]. Studies of Italian bitterns also showed that frequency-based features of bittern booms vary over time, and re-identification can be difficult [12].

In areas where vocal individuality is not used, bitterns have been counted using acoustic surveys of calling individuals. In low-density populations, individuals show high site fidelity [53] therefore simply counting calling individuals may be adequate. However, populations at higher densities, where individuals compete more strongly for resources, are likely to contain a number of non-territorial individuals that as a consequence are mobile and vocally active (floaters). Unless these individuals are accounted for they can cause large over-estimates of population size [39].

#### Corncrake

Corncrakes are a species of land rail that primarily inhabit hay meadows and silage fields [41]. Although population numbers in the UK have increased in recent years due to increased habitat management (currently estimated at 600–700 calling males [111]), they are listed as a species of global conservation concern (IUCN red-listed, [108]) and are on Annex 1 of the EU Wild Birds Directive [109]. As the species is secretive and nocturnal, the only viable method to census a population is by counting calling males in the breeding season. Currently 90% of the UK habitat for corncrakes is monitored in this manner [109].

The census strategy used for corncrakes was developed from radio-tagging studies [41], which found that males rarely move more than 250 m between nights and that on any particular night around 75% of males would call. Based on these findings, censuses were carried out on two nights and if two calling locations were within 250 m of each other on both nights, they were judged to come from the same individual [41]. Corncrakes have individually distinctive vocalizations [13,112], which are consistent over years [13]. The census rules present obvious sources of bias and Peake & McGregor [40] monitored corncrakes using vocal individuality to show that males called far less than anticipated (41% of males per night) and this led to the population being underestimated by up to 30%. They were also able to follow individuals throughout the season and showed that habitat quality affected movement, with males in poor quality habitat moving greater distances. Their study also shows that tracking movements using the standard census approach was in most cases accurate [40]. The main role for vocal individuality in this case would be to provide correction factors to refine standard census rules or as a method for monitoring individual movements in relation to small-scale habitat differences.

#### Owl species

Owls, like most raptors, occur at low densities and are usually difficult to monitor. Most owl species are territorial and show high site fidelity after dispersal. However, monitoring territory occupancy, survival and recruitment can be difficult for these mostly nocturnal and secretive species. However, most owl species are characterised by their long-range vocalizations and these have been shown to be individually distinctive for several species (e.g. barred owls, *Strix varia *[113]; Northern saw whet owls, *Aegolius arcadicus *[114]; eagle owls, *Bubo bubo *[115]; screech owls, *Otus asio *[116]; Christmas Island hawk owls, *Ninox natalis *[117]; tawny owls, *Strix aluco *[75,118]; pygmy owls, *Glaucidium passerinum *[76]; African wood owls, *Strix woodfordii *[77] European scops owls, *Otus scops *[54] and Seychelles scops owls (*Otus insularis *[Peake, Currie & McGregor pers comm.]). These vocalizations seem to remain stable during a season and, where studied, were consistent for long periods of time [54,77,115].

Vocal individuality has proved to be a useful tool for mapping territories in these species and monitoring habitat use through the breeding season. Galeotti & Sacchi [54] showed a high annual turnover on breeding territories for European scops owls, with 55–78% of territories occupied by different individuals between years (note that vocal individuality was validated by previous studies in this species). They suggest that this high turnover may be related to their migratory habits and possible high winter mortality, which leaves many vacant territories at the beginning of the breeding season [54]. African wood owls are a sedentary species where both sexes call. They form monogamous pairs that maintain stable territories. Delport et al [77] recorded wood owls at different calling locations over a 12-year period to monitor territory occupancy and turnover for each sex. They found a turnover of 19.3% and 13.7% for males and females respectively. This is one of the few examples where both sexes can be monitored with vocal individuality.

In both the previously mentioned cases [54,77], individuals were not independently marked, and vocal stability is assumed based on previous studies of other owl species [75,76] and the presence of temporal stability in the call features used [77]. However, this assumption of vocal stability has to be treated with care, as if a vocalization changes between years, it will not be possible to determine whether the individual or the vocalization is different.

### Potential and limitations of vocal individuality

We have explained how vocal individuality can have a role in conservation and we have presented case studies in which it has been used to generate useful data. For such a potentially useful technique, vocal individuality is surprisingly under-utilised. Our case studies show a heavy bias towards male territorial signalling in avian species. This bias is a natural consequence of an avian bias in bioacoustics and communication generally and the fact that territorial signals are the most obvious. However, we feel that there are other classes of individual and other taxa that could be represented in vocal individuality studies.

In this section we discuss some of the potential limitations of vocal individuality (concentrating particularly on vocal stability and sex differences in vocal activity), we indicate the sort of information required to assess its potential as a conservation tool and we explore the potential of vocal individuality in mammals.

#### Some limitations of using vocal individuality

All identification techniques contain a number of potential biases and disadvantages that need to be borne in mind in order to minimise the chance of producing misleading results or limiting their explanatory power. Four main problems are encountered when using vocal individuality. First, establishing the extent and stability of individuality requires intensive study with, preferably, independently marked individuals. Second, this intensive study requires knowledge of sound analysis and the equipment used for recording and analysis. Third, vocal individuality is biased towards the most vocally active sections of the population (and there are similar biases in any method based on acoustics). Many factors can affect which portion of a population is vocally active, for example sex (e.g. it is the males of most temperate bird species that are vocally active), age, time of year, breeding status, territorial status (e.g. great horned owls, *Bubo virginianus *[55]) or dominance. For vocal individuality to provide useful information, these sub-groups have to be known. Fourth, because vocal individuality uses natural variation, there will always be a level of ambiguity in the identification of an individual [2]. We consider the most important current limitations on the value to conservation of vocal individuality to be vocal stability and monitoring females, so we shall discuss these topics in more detail.

##### Vocal stability over time

Vocal stability over time is difficult to show because ideally it requires independent identification of individuals. The case studies involving corncrakes and bitterns (see above) both used a sample of radio-tagged males to collect recordings over time [12,41,53]. With the exception of Lengagne [115], studies involving owls have looked for temporal stability in vocal features with time as an indication of vocal stability [54,77]. They have also used previous studies showing long-term vocal stability in different owl species to support their assumptions [75,76], although none of those individuals were independently marked. When using vocal individuality to re-identify individuals, for example when monitoring populations, establishing vocal stability needs special consideration and, as with bitterns (see above) may require an increase in effort, equipment and knowledge. Such extra effort may limit the applicability of vocal individuality in monitoring contexts.

##### Monitoring females

For many species, especially avian, monitoring the presence and movement of females will be difficult without radio-tracking. For example females of most temperate avian species are quiet and do not have long-range vocalizations. Exceptions to this include some non-oscine species such as petrels and some owl species. Thus in many cases population estimates are reported as the number of calling males rather than pairs (e.g. corncrakes and bitterns [108]). Indirect measures of female presence are sometimes possible, for example male corncrakes cease calling for some days when they have attracted a mate [13]. There are also exceptions, for example, female brown-headed cowbirds (*Molothrus ater*) have a loud long-range individually distinctive call that can be used to identify them [119]. As mentioned above, male and female wood owls have long-range territorial vocalizations [77]. In cases where females do not have obvious long-range vocalisations, it may be possibly to exploit calls used to maintain contact between pairs, with offspring or with other social group members as a way of identifying and tracking females. For example, Campbell et al [97] showed that female Stellar sea lions could be identified from their mother-pup calls.

Colonial breeding species, such as many species of seabird and marine mammal, rely on contact calls (and other cues) to locate their mates and offspring [6,97,120,121]. As such, the vocalisations of these species have been shown to contain high levels of individuality. In most cases, both the males and females vocalise, often with discriminable sex differences. Information from individuality could be combined with other census methods involving calls for these species, for example many species of petrel are burrow-nesting and nocturnal, however, they possess extensive vocal repertoires [122]. These species also readily respond to playback of calls and this method is used to census populations and to determine burrow occupation [123-125]. In some species, primarily tropical and monogamous, duetting is common between pair-bond members, and calls become more similar between the male and female as the bond develops with increasing time (e.g. gibbon species [126]; red-fronted parrots, *Poicephalus gulielmi *[127]; twites, *Acanthus flavirostris *[128]. Although this may make the discrimination task more difficult, it may yield information on pair identity when the individuals are separated and the length of the pair bond.

In species with more complex social systems females are also vocally active, and often both sexes can be discriminated by individual and gender [129]. Female chacma baboons (*Papio ursinus*) have individually distinctive contact and alarm calls [130] and Weiss et al [131] showed that cotton-top tamarin (*Saguinus oedipus*) calls contain information on individual, sex and group identity. In several social canid species, females are as vocally active as males and take part in long-range vocalizing [132-136]. Vocalizations have been found to be individually distinctive for many of these species [78,132-134]. However, the rate of vocalizing in some canid species is related to social dominance, season, and whether they are transient or resident groups [135,136], and monitoring strategies would have to take account of this.

#### Vocal individuality as an effective conservation tool

Most of the literature suggesting that vocal individuality has a role in conservation is in fact represented by studies of the presence of vocal individuality and not whether it provides an effective conservation tool. To provide an indication of effectiveness as a conservation tool requires information on several topics and consideration of several issues. We consider the following to be most important.

1) The vocalization used should be easily recordable and provide the best potential for vocal individuality. It is generally considered that long-range advertising vocalizations most readily fulfil these criteria [65]. Species with a repertoire of such vocalizations may present the problem of which vocalization to use for individual identification. The problem can be addressed by either choosing one vocalization (such as the most common and/or distinctive) or by looking at features common to all vocalizations in the repertoire (e.g. [137,138]).

2) The sample size tested should be similar to the number of individuals that will be discriminated when the technique is used. In many cases vocal individuality studies have used smaller sample sizes (10–20 individuals) than if the technique were to be used as a conservation tool.

3) Careful consideration is needed in choosing which measures to take and every effort should be made to pare them down (using principle components analysis or stepwise discriminant analysis) to the most effective discriminators. The best measures will be those that can be extracted from recordings of varying quality. Some techniques standardize recording quality by only accepting those recordings that include specific sections of the signal, usually of lower amplitude, for analysis [53]. Atmospheric conditions affect temporal, amplitude and frequency components of a signal in different ways [139]. Different analysis types are also affected by recording quality, for example the instantaneous frequency of bittern booms can discriminate between individuals even in poor quality recordings [110]. However, if temporal measures are to be taken from a waveform display, recordings have to be of a higher quality because background noise will mask the signal.

4) When multivariate statistical tools are used to discriminate and identify individuals, we recommend the use of similarity techniques (see above) as they do not need the population size to be known and they also allow for the identification of additional individuals.

5) Any application of vocal individuality will not use recordings in isolation to identify individuals. A lot of data can be collected at the time of recording that will reduce the number of individuals that have to be discriminated. The person making the recording can note other simultaneously calling individuals and the locations and times of all recordings. This can be important if a technique seems to lack discrimination power.

6) The most effective test of vocal individuality will be through simulated census and monitoring situations [81,98]. These can be achieved through blind trials and repeated random sampling from known data sets to create population samples of unknown size and composition. We tested the use of neural network models in census and monitoring tasks by using a data set of 30 individuals that was randomly sampled [98]. The same individual could appear several times, and we used neural networks to classify individuals in a series of blind trials. These kinds of test more accurately simulate how the technique will perform as a conservation tool.

7) In most cases the people developing and testing monitoring techniques are not the same as those who will be using them in the field. Thus, one important, if obvious, point is that specific guidelines need to be given to those who will collect and analyse recordings. For many endangered or low-density populations, collecting recordings requires considerable fieldwork (e.g. [53]), and therefore this warrants the most efficient (effort vs. results) analysis possible. It may not be possible to rely on spontaneous vocalizing to collect enough recordings and playback is often used to elicit calls. However, the time of playback or its overuse can also have biasing effects, for example by causing individuals to move off their territories [37,38].

#### Vocal individuality in mammals

As demonstrated by the examples we have used in this review, applications of vocal individuality have been limited to avian species, usually vocally active species that are difficult to monitor with conventional methods. A notable exception is the study of swift foxes (*Vulpes velox*) by Darden et al [79]. Many other mammalian species are vocally activity on land and are difficult to monitor conventionally because they occur at low densities in dense habitat. In addition, many have a rich vocal repertoire, with both sexes vocalizing or with vocal communication between parents and offspring. However, the general structure of the mammalian vocal system causes not only complex signal structures (e.g. harmonics) but also modifications (e.g. biphonation and chaotic noise), making it more difficult to analyse (but see [79]). Perhaps more importantly, the signalling context can have large effects on the structure of the sound produced, which can affect its use in identification [140]. This seems to be a common phenomenon in primates. For example, the degree of vocal variability in chimpanzees is related to the amount of social chorusing between males, with the amount of time males spent together increasing the similarity of their calls [140,141]. Several marmoset species show individually distinctive vocalizations when either isolated [142] or in stable social groups, but in novel social conditions the vocal structures change [143]. Despite these potential problems, the number of reports of vocal individuality in mammals indicates that it could be a useful conservation tool (e.g. [144]).

For cetaceans, the underwater acoustic channel is the most important means of communication [145,146]. Studying individual vocal differences in cetaceans and other aquatic mammals has proved to be difficult due to problems in identifying which individual is calling, with the result that most studies have been at the group or population level [145]. More recently, various techniques have been used to locate individual callers either with theodolites, hydrophone arrays, or tags [146]. Also individuals have been identified over longer periods of time using photo-identification, mostly of dorsal fin [147] or tail-fluke features [148]. However, visual identification techniques are less readily applied when large groups of individuals are together [146]. Few studies have been made of individually distinctive features of their vocal behaviour, and none have used this individuality to follow individuals.

The most studied cetaceans are dolphins. Bottle-nose dolphins (*Tursiops truncatus*) have individually distinctive signature whistles [149-151], and these whistles can remain constant for over a decade [151,152] but they can change depending on the social context [153,154], and individuals are capable of copying the whistles of others [152] which then may be used in matched calling encounters [155]. Signature whistles would seem to be ideal for vocal individuality; however, social effects present several potential problems in their use. Also, the use of signature whistles may decrease when individuals are in groups [154]. It remains to be seen whether acoustic signals can be used to monitor dolphin species.

Large cetaceans are capable of communicating over large distances and are acoustically active, especially during the breeding season [145]. These acoustic signals have the ability to generate information on group identity, body size and interactions [146]. Studying cetaceans in stable social groups has revealed the more complex aspects of communication. Both sperm whales (*Physeter macrocephalus*) and killer whales (*Orcinus orca*) produce both individually and group distinctive signals [107,156,157]. The structure of sperm whale clicks also gives information about the size of the caller [158]. Few studies have used individuality to follow individuals (but see Fig. 10 in [152]).

## Conclusion

There will be many instances where vocalizations are the only evidence of the presence of members of a population. Often, these populations are of conservation concern and baseline demographic information is difficult to obtain. Identifying individuals using individually distinctive vocalizations offers an alternative to marking that avoids problems associated with handling and sampling biases. As with other monitoring techniques, vocal individuality contains biases that have to be accounted for when it is used. Vocal individuality is not a panacea to all monitoring problems, but where a species is sensitive to disturbance or difficult to monitor (either through its behaviour or because of its environment), utilising its vocal behaviour can provide an effective conservation tool.

## Authors' contributions

AMRT carried out the initial literature survey and drafted the first version of the manuscript. All authors expanded and developed the review. PKM produced the final version of the manuscript which was read and approved by all authors.
